# Genotypic Diversity and Population Structure of *Vibrio vulnificus* Strains Isolated in Taiwan and Korea as Determined by Multilocus Sequence Typing

**DOI:** 10.1371/journal.pone.0142657

**Published:** 2015-11-23

**Authors:** Hye-Jin Kim, Jae-Chang Cho

**Affiliations:** 1 Institute of Environmental Sciences and Department of Environmental Sciences, Hankuk University of Foreign Studies, Yong-In, Korea; 2 Solana Informatics, San Diego, CA, United States of America; Columbia University, UNITED STATES

## Abstract

The genetic diversity and population structure of *Vibrio vulnificus* isolates from Korea and Taiwan were investigated using PCR-based assays targeting putative virulence-related genes and multilocus sequence typing (MLST). BOX-PCR genomic fingerprinting identified 52 unique genotypes in 84 environmental and clinical *V*. *vulnificus* isolates. The majority (> 50%) of strains had pathogenic genotypes for all loci tested; moreover, many environmental strains had pathogenic genotypes. Although significant (*p* < 0.05) inter-relationships among the genotypes were observed, the association between genotype and strain source (environmental or clinical) was not significant, indicating that genotypic characteristics alone are not sufficient to predict the isolation source or the virulence of a given *V*. *vulnificus* strain and vice versa. MLST revealed 23–35 allelic types per locus analyzed, resulting in a total of 44 unique sequence types (STs). Two major monophyletic groups (lineages A and B) corresponding to the two known lineages of *V*. *vulnificus* were observed; lineage A had six STs that were exclusively environmental, whereas lineage B had STs from both environmental and clinical sources. Pathogenic and nonpathogenic genotypes predominated in MLST lineages B and A, respectively. In addition, *V*. *vulnificus* was shown to be in linkage disequilibrium (*p* < 0.05), although two different recombination tests (PHI and Sawyer’s tests) detected significant evidence of recombination. Tajima’s D test also indicated that *V*. *vulnificus* might be comprised of recently sub-divided lineages. These results suggested that the two lineages revealed by MLST correspond to two distinct ecotypes of *V*. *vulnificus*.

## Introduction

*Vibrio vulnificus* is a halophilic, Gram-negative, curved, rod-shaped bacterium frequently found in marine environments (e.g., estuarine and coastal waters) as well as in molluscan shellfish (e.g., oysters and clams) as part of the normal microflora [1–3]. *V*. *vulnificus* is also an important human pathogen, infection by which mainly occurs through the consumption of contaminated seafood and occasionally through open wounds exposed to contaminated seawater [1, 3, 4]. Although most data regarding the epidemiology of *V*. *vulnificus* infection are for the United States of America (USA) [2, 5–8], human infections with *V*. *vulnificus* have also been reported in many other countries [9–16]. The most fatal consequence of *V*. *vulnificus* infection is primary septicemia, which has a mortality rate of over 50% in immunocompromised individuals [2, 5, 17]. Due to the high mortality rate of *V*. *vulnificus* infection, *V*. *vulnificus* contamination is a serious food safety issue in regions where seafood (especially raw oysters) consumption is common. Until recently, studies of *V*. *vulnificus* focused mainly on the virulence factors that confer its remarkable pathogenicity. The *V*. *vulnificus* virulence factors identified to date include capsular polysaccharide (CPS) [18, 19], lipopolysaccharide (LPS) [20, 21], hemolysin [22, 23], the RtxA1 toxin [24, 25], and other molecules involved in iron acquisition and the formation of pili and flagella [26–29]. To evaluate and predict the virulence of different *V*. *vulnificus* strains, several genotyping methods have been developed [30–38]. Although genotyping studies are not a substitute for animal models for evaluating the virulence of *V*. *vulnificus* strains, such studies provide valuable information regarding the distribution of *V*. *vulnificus* genotypes.

Since most cases of *V*. *vulnificus* infection have been reported primarily in the Gulf Coast region of the USA [39–42] and in specific regions of Asia [10, 43–45], environmental factors, geographic factors, and seafood consumption patterns of inhabitants in these regions could all play an important role in *V*. *vulnificus* epidemiology. Interestingly, *V*. *vulnificus* infection is most prevalent in temperate coastal regions [3, 46, 47]. Moreover, the majority (>70%) of *V*. *vulnificus* infections in Korea occur on the southwest coast, whereas *V*. *vulnificus* infection is very rare on the east coast [48–50]. This is a striking observation, since the dietary preferences of the inhabitants of the southwest coast and the east coast are quite similar. Additionally, in Taiwan, most reported cases (>90%) of *V*. *vulnificus*-induced illness have occurred in the southern part of Taiwan [10]. The prevalence of virulent strains might vary by geographic region, although the proportion of susceptible individuals might also vary within a region according to socioeconomic status or other factors [6].

Extensive studies of *V*. *vulnificus* virulence factors have been performed using highly virulent strains (CMCP6, MO6-24/O, and YJ016) isolated in Korea, the United States, and Taiwan [51–53]. However, the natural populations of *V*. *vulnificus* have been poorly characterized, especially the *V*. *vulnificus* populations found in Asian countries. The genotypic distribution patterns of *V*. *vulnificus* have been investigated mainly in the Gulf Coast region [54–57], while other studies have examined the genetic diversity of *V*. *vulnificus* in the northeastern United States and in European countries [35, 36, 58, 59]. In this study, we investigated the genetic diversity of *V*. *vulnificus* strains isolated in Taiwan and Korea. These strains were subjected to PCR-based genotypic characterization of putative virulence factors and to multilocus sequence typing (MLST) using seven genetic loci. The association between the genotypic characteristics of *V*. *vulnificus* strains and their source of origin, as well as the population structure of *V*. *vulnificus* as inferred from the MLST data, are reported here.

## Materials and Methods

### Bacterial strains, biochemical tests, and general molecular techniques

A total of 84 strains of *V*. *vulnificus* (hereafter referred to as VV) originating from the United States, Taiwan, and Korea were included in this study (Table 1). Clinical strains (n = 21) had been previously isolated from hospitalized patients with septicemia [51–53, 60–64], whereas environmental strains (n = 63) were isolated from seawater, seafood, and tidal mudflat samples. Three clinical strains whose genomes had been sequenced (CMCP6, MO6-24/O, and YJ016) [51, 53, 64] were used as reference strains. All VV strains were routinely cultured in Luria-Bertani medium, either with or without 2.5% NaCl (LB or LBS), at 37°C. Frozen stocks were stored at -80°C in LBS with 70% (vol/vol) glycerol.

**Table 1:** *Vibrio vulnificus* strains used in this study.

Strain	Isolation source^a^	Country of isolation	Reference^b^	Strain	Isolation source^a^	Country of isolation	Reference^b^
ATCC 29307	C	United States	70, ATCC	SC9613	E (crab)	Korea	58, 59
CG108	E	Taiwan	60	SC9624	E (shell)	Korea	58, 59
CG110	E	Taiwan	60	SC9629	E (clam)	Korea	58, 59
CG122	E	Taiwan	60	SC9631	E (clam)	Korea	58, 59
CG21	E	Taiwan	60	SC9641	E (octopus)	Korea	58, 59
CG26	E	Taiwan	60	SC9648	E (clam)	Korea	58, 59
CG27	E	Taiwan	60	SC9649	E (shell)	Korea	58, 59
CG33	E	Taiwan	60	SC97100	E (conch)	Korea	58, 59
CG46	E	Taiwan	60	SC97114	E (tidal mudflat)	Korea	58, 59
CG54	E	Taiwan	60	SC97116	E (crab)	Korea	58, 59
CG55	E	Taiwan	60	SC97118	E (tidal mudflat)	Korea	58, 59
CG57	E	Taiwan	60	SC97126	E (oyster)	Korea	58, 59
CG58	E	Taiwan	60	SC9716	E (seawater)	Korea	58, 59
CG62	E	Taiwan	60	SC9717	E (tidal mudflat)	Korea	58, 59
CG64	E	Taiwan	60	SC9720	E (tidal mudflat)	Korea	58, 59
CNUH94-6	C	Korea	58, 59, CNUH	SC9721	E (seawater)	Korea	58, 59
CMCP6	C	Korea	68	SC9728	E (seawater)	Korea	58, 59
CN7	C	Korea	58, 59, CNUH	SC9729	E (seawater)	Korea	58, 59
CN8	C	Korea	58, 59, CNUH	SC9730	E (tidal mudflat)	Korea	58, 59
CN9	C	Korea	58, 59, CNUH	SC9731	E (seawater)	Korea	58, 59
CNUH94-3	C	Korea	58, 59, CNUH	SC9733	E (fish)	Korea	58, 59
CNUH94-4	C	Korea	58, 59, CNUH	SC9737	E (tidal mudflat)	Korea	58, 59
CS91133	C	Korea	58, 59, CNUH	SC9738	E (oyster)	Korea	58, 59
MO6-24/O	C	United States	67	SC9740	E (seawater)	Korea	58, 59
NV1	E	Taiwan	60	SC9761	E (oyster)	Korea	58, 59
NV101	E	Taiwan	60	SC9763	E (seawater)	Korea	58, 59
NV15	E	Taiwan	60	SC9766	E (clam)	Korea	58, 59
NV18	E	Taiwan	60	SC9771	E (shell)	Korea	58, 59
NV22	E	Taiwan	60	SC9793	E (seawater)	Korea	58, 59
NV24	E	Taiwan	60	SC9794	E (tidal mudflat)	Korea	58, 59
NV28	E	Taiwan	60	SC9795	E (seawater)	Korea	58, 59
NV31	E	Taiwan	60	V-15	C	Korea	58, 59, CNUH
NV33	E	Taiwan	60	V-16	C	Korea	58, 59, CNUH
NV37	E	Taiwan	60	V-19	C	Korea	58, 59, CNUH
NV42	E	Taiwan	60	WK13	C	Korea	58, 59, WKUH
NV43	E	Taiwan	60	WK15	C	Korea	58, 59, WKUH
NV55	E	Taiwan	60	WK16	C	Korea	58, 59, WKUH
NV61	E	Taiwan	60	WK20	C	Korea	58, 59, WKUH
NV63	E	Taiwan	60	WK22	C	Korea	58, 59, WKUH
NV69	E	Taiwan	60	WK3	C	Korea	58, 59, WKUH
NV72	E	Taiwan	60	WK6	C	Korea	58, 59, WKUH
NV78	E	Taiwan	60	YJ016	C	Taiwan	69

^a^ C and E denote clinical (human patient with septicemia) and environmental (seawater, seafood, and tidal mudflat) sources, respectively.

^b^ Literature or additional information (e.g., strain provider). ATCC, American Type Culture Collection; CNUH, Chonnam National University Hospital, Korea; WKUH, Won Kwang University Hospital, Korea. i, Hollis et al. [61]; ii, Wong et al. [123]; iii, Kim et al. [62] and Lee et al. [63]; iv, Kim et al. [51]; v, Wright et al. [52]; vi, Shao and Hor [60].

VV strains were biotyped using the following biochemical tests: i) indole production from tryptophan (indole reaction), ii) putrescine production from orinithine (ornithine decarboxylation, ODC reaction), and iii) o-nitrophenol production from o-nitrophenyl-β-D-galactopyranoside (ONPG reaction). Biotypes were assigned according to the methods of Tison et al. [65] and Bisharat et al. [66] (biotype 1, positive for indole and ODC reactions; biotype 2, negative for indole and ODC reactions; biotype 3, ONPG-negative).

Genomic DNA was extracted from bacterial cultures in exponential growth phase using Genomic-Tip kits (Qiagen, Valencia, CA, USA) according to the manufacturer's protocol and stored in sterile water at -20°C until use. Unless specified otherwise, all other general experimental procedures were performed according to a standard laboratory manual [67].

### BOX-PCR genome fingerprinting

Repetitive extragenic palindromic PCR (rep-PCR) genomic fingerprinting of the VV strains was carried out with a BOX-A1R primer (BOX-PCR) according to the protocol of Cho and Tiedje [68], with minor modifications. Similarity matrices of whole densitometric curves of the gel tracks were calculated using the pairwise Pearson's product-moment correlation coefficients (*r* values) [69] as described by Rademaker et al. [70]. Cluster analyses of the similarity matrices were performed using the unweighted pair group method with arithmetic averages (UPGMA).

### Genotypic characteristics

Representative strains that were selected based on the UPGMA clustering of their BOX-PCR fingerprinting patterns were subjected to genotype analyses. Six genetic loci, *pilF* (pilus-type IV assembly gene), *vcg* (ORF no. VV0401, virulence-correlated gene of strain YJ016), *viuB* (vulnibactin gene), *vuuA* (ferric vulnibactin receptor), *vvhA* (VV hemolysin gene), and CPS (capsular polysaccharide) alleles were analyzed according to the methods of Roig et al. [30], Rosche et al. [71], Panicker et al. [72], Kim et al. [73], Kaysner and DePaola [74], and Han et al. [75], respectively. Associations between genotypic characteristics and strain origin (environmental or clinical), as well as associations among the genotypic characteristics themselves, were evaluated with the chi-square (χ^2^) test and Fisher's exact test (α = 0.05).

### Multilocus sequence typing (MLST)

Representative strains of BOX-PCR types (Table 2) were subjected to MLST of seven genetic loci. These loci included six housekeeping genes that encoded glutamine synthetase (*glnA*), glucose-6-phosphate isomerase (*glp*), DNA gyrase subunit B (*gyrB*), malate-lactate dehydrogenase (*mdh*), dihydroorotase (*pyrC*), and recombinase A (*recA*), in addition to a virulence-associated gene encoding VV hemolysin (*vvhA*) (details described in S1 Table). *vvhA* was included to supplement the phylogenetic information collected from the housekeeping genes with information from a VV-specific gene. Partial sequences of the MLST loci were obtained using specific primer pairs and the amplification conditions described in the PubMLST database [76] for *glp*, *mdh*, and *pyrC*; by the conditions described by Gutacker et al. [77] for *glnA* and *recA*; and by the conditions described by Kotetishvili et al. [78] for *gyrB*. PCR amplicons were sequenced using an ABI3700 DNA analyzer (Applied Biosystems, Foster City, CA, USA). DNA sequences of each gene were aligned with CLUSTAL W [79]. No gaps (indels) were introduced into any of the alignments, and no manual editing was performed.

**Table 2:** Genotypic characteristics of representative *Vibrio vulnificus* strains as assessed by BOX-PCR genomic fingerprinting.

BOX-PCR profile no.^a^	Representative strain^b^	No. of strains^c^	Soruce of isolation^d^	16S rRNA type^e^	CPS type^e^	*vcg* type^e^	*pilF* ^e^	*viuB* ^e^	*vuuA* ^e^
1	SC9629	2	E	A	NA^f^	E	**-**	**-**	**-**
2	NV22	2	E	A	NA	C	**-**	**-**	**-**
3	SC9729	1	E	A	2	E	**-**	**-**	**-**
4	SC9740	1	E	A	NA	E	**-**	**-**	**-**
5	SC9613	1	E	A	NA	E	**+** ^**g**^	**-**	**-**
6	CNUH94-4	1	C	B	1	C	**+**	**-**	**+**
7	V-16	1	C	B	2	C	**+**	**-**	**+**
8	CN8	2	C	B	1	C	**+**	**+**	**+**
9	CN7	1	C	B	1	C	**+**	**+**	**+**
10	YJ016	2	C	B	2	C	**+**	**+**	**+**
11	SC9733	1	E	B	1	C	**+**	**-**	**+**
12	CG122	1	E	B	NA	C	**+**	**+**	**-**
13	CG55	2	E	B	2	C	**+**	**+**	**+**
14	CG108	1	E	B	1	C	**+**	**+**	**+**
15	SC9648	1	E	B	2	C	**+**	**-**	**+**
16	SC9720	1	E	B	2	C	**+**	**-**	**+**
17	SC9761	1	E	A	2	E	**+**	**-**	**+**
18	NV63	2	E	B	2	C	**+**	**+**	**+**
19	SC9737	1	E	B	1	C	**+**	**+**	**+**
20	NV72	2	E	B	2	C	**+**	**+**	**+**
21	CG27	4	E	B	1	C	**+**	**-**	**+**
22	CG26	2	E	B	1	C	**+**	**+**	**+**
23	SC97118	4	E	B	2	C	**+**	**+**	**+**
24	WK22	2	C	B	1	C	**+**	**+**	**+**
25	NV101	1	E	B	NA	C	**-**	**-**	**-**
26	WK20	4	C	B	1	C	**+**	**+**	**+**
27	V-19	1	C	B	1	C	**+**	**+**	**+**
28	SC9717	1	E	B	1	C	**+**	**+**	**-**
29	CG33	1	E	B	1	C	**+**	**-**	**+**
30	CG62	2	E	B	2	C	**+**	**+**	**+**
31	NV42	1	E	B	1	C	**+**	**+**	**+**
32	CG21	1	E	B	1	C	**+**	**+**	**+**
33	SC9794	1	E	B	1	C	**+**	**-**	**+**
34	NV37	3	E	B	1	C	**-**	**+**	**-**
35	SC9730	1	E	B	2	C	**+**	**+**	**-**
36	SC9649	2	E	B	2	C	**+**	**+**	**+**
37	CNUH94-3	1	C	B	1	C	**+**	**-**	**+**
38	SC9721	1	E	B	2	C	**+**	**+**	**-**
39	NV43	1	E	B	1	C	**+**	**+**	**-**
40	NV28	3	E	B	NA	C	**+**	**+**	**-**
41	NV31	2	E	B	NA	C	**+**	**-**	**-**
42	NV18	1	E	B	NA	C	**+**	**+**	**-**
43	SC9766	3	E	B	NA	C	**+**	**-**	**-**
44	WK15	2	C	B	1	C	**+**	**+**	**+**
45	WK6	1	C	B	1	C	**+**	**+**	**+**
46	CG64	1	E	B	1	C	**+**	**+**	**+**
47	NV1	3	E	B	1	C	**+**	**+**	**+**
48	SC9793	1	E	B	1	C	**+**	**+**	**+**
49	MO6-24/O	2	C	B	1	C	**+**	**+**	**+**
50	ATCC29307	1	C	B	2	C	**+**	**-**	**+**
51	CMCP6	1	C	B	1	C	**+**	**-**	**+**
52	WK13	2	C	B	1	C	**+**	**-**	**+**

^a^ As shown in Fig 1.

^b^ Selected randomly, with the exception of the reference clinical strains CMCP6, MO6-24/O, and YJ016.

^c^ Number of strains belonging to each BOX-PCR profile.

^d^ C, clinical source; E, environmental source.

^e^ Determined according to the methods of Nilsson et al. [37], Han et al. [75], Roche et al. [71], Roig et al. [30], Panicker et al. [72], and Kim et al. [73].

^f^ Not amplified.

^g^ PCR amplicon with the expected size.

The seven multiple sequence alignments were concatenated, and phylogenetic reconstruction was performed from the concatenated sequence of the MLST loci. A phylogenetic tree was inferred from the concatenated sequence using the neighbor-joining (NJ) algorithm implemented in MEGA software (ver. 5) [80]. The evolutionary distances between the sequences were calculated according to the Jukes-Cantor (JC69) substitution model [81]. The tree topology was statistically evaluated by 1,000 bootstrap resamplings and was further confirmed using the maximum likelihood (ML) algorithm (general time reversal [GTR] + gamma [Γ] distribution model) implemented in the high performance computing version of RAxML [82]. Phylogenetic analyses were also performed for individual genes, as described above, to determine the alleles of each gene (S1 Fig).

An allelic type (AT) was defined as a unique combination of polymorphisms within a gene. Each AT was assigned an arbitrary number. A sequence type (ST) was defined as a unique combination of ATs within the concatenated sequences (seven genes combined) according to START (ver. 2) [83]. Each ST was also assigned an arbitrary number.

### Tests of neutrality, linkage disequilibrium, and recombination

From the ATs, a number of descriptive properties were determined for each gene. The number of polymorphic sites, the number of parsimonious sites, the nucleotide diversity (π, average pairwise nucleotide difference per site) [84], the average number of nonsynonymous substitutions per nonsynonymous site (dN), the average number of synonymous substitutions per synonymous site (dS), and Tajima's D statistic [85] for each gene were calculated using DNASP (ver. 5.1) [86]. To investigate whether positive (or negative) selection had occurred at the protein level, the ratio of dN to dS was calculated (dN/dS < 1, negative selection; dN/dS = 1, no selection; dN/dS > 1, positive selection). Tajima's D statistic was used to test the neutrality of the observed DNA polymorphisms under the assumption that the Tajima D values exhibit a β distribution (D < 0, high level of low frequency polymorphisms compared with the expected level in a neutral model [possibly due to population size expansion after a bottleneck or selective sweep]; D > 0, low level of polymorphisms compared with the expected level [possibly due to population size contraction or balancing selection]; D = 0, observed level of polymorphisms similar to the expected level [possibly due to genetic drift-mutation equilibrium]) [85, 87].

Multilocus linkage disequilibrium between alleles (nonrandom association of alleles at multiple loci) [88] was examined by determining the index of association (I_A_ ≠ 0, disequilibrium; I_A_ = [V_O_/V_E_] - 1, where V_O_ = observed variance and V_E_ = expected variance) using START (ver. 2) [83]. Since I_A_ depends on sample size (i.e., the number of loci used), the standardized version of I_A_ (I^S^_A_ = [V_0_/V_E_—1]/[L-1], where L = the number of loci) [89] was also determined. Statistical significance levels of I_A_ and I^S^_A_ were measured using a randomization test with 1,000 iterations.

Recombination analyses for the genes analyzed by MLST were performed according to the method described by Martin et al. [90]. The overall evidence of recombination at each locus was assessed by determining the Φ statistic (pairwise homoplasy index, PHI) [91]. The PHI test detects recombination by comparing the frequency of phylogenetically incompatible site-pairs with the frequency of such site pairs expected in the absence of recombination [90]. The PHI test was performed with SPLITSTREE (ver. 4.13) [92]. Window size (W) was set to 100 bases (Φ_W = 100_), and the statistical significance of Φ_W_ values were assessed using a permutation test with 1,000 iterations (H_0_ [no recombination, Φ_W_ ≠ 0] rejected at α = 0.05 in favor of H_1_ [recombination]), as recommended by Bruen et al. [91]. Individual recombination events for each locus were inferred from evidence of gene conversions (fragments of DNA sequence that are copied onto another fragment of DNA sequence) [93] as detected by Sawyer's runs test [94]. This test identifies the DNA fragment shared by two sequences via ancestral gene conversion and was performed using GENECONV (www.math.wustl.edu/~sawyer). The statistical significance of detecting the shared fragment (intragenic conversion) was measured using a permutation test with 10,000 iterations. A single recombination event was defined as a group of fragments linked to the same 5' and/or 3' breakpoints, as described by Nightingales et al. [95] and den Bakker et al. [96]. After statistical assessment of recombination, all ST sequences were used to generate a Neighbor-Net network [97] tree depicting the evolutionary relationships between VV strains (ST sequences) with ancestral recombination events [90]. The Neighbor-Net network based on weighted splits in multiple sequence alignment was constructed using SPLITSTREE (ver. 4.13) [92]. The JC69 substitution model [81] used in the bifurcating phylogenetic tree was used to calculate the evolutionary distances in the Neighbor-Net network, and the network topology was evaluated by 1,000 bootstrap resamplings. All other calculations were performed according to the SPLITSTREE manual.

### Nucleotide sequence accession numbers

The nucleotide sequences of the 16S RNA gene, *glnA*, *glp*, *gyrB*, *mdh*, *pyrC*, *recA*, and *vvhA* have been deposited at NCBI GenBank under the accession numbers KP223857-KP223908, KP223909-KP223960, KP223961-KP224012, KP224013-KP224064, KP224065-KP224116, KP224117-KP224168, KP224169-KP224220, and KP224221-KP224272, respectively.

## Results and Discussion

### Collection of biotype 1 strains

Consistent with the finding that biotype 1 strains have been primarily identified as responsible for human infection [1, 2], all clinical strains in our collection belonged to biotype 1. However, all of our environmental strains also belonged to biotype 1, regardless of the isolation source (e.g., seawater, seafood, or tidal mudflat). Unlike the biotype 1 strains, which have been found worldwide in the marine environment (including brackish water) as well as in patients with septicemia [2], VV strains belonging to biotypes 2 and 3 have been reported to occupy specialized ecological niches. Biotype 2 strains were primarily found as eel (genus *Anguilla*) pathogens [65, 77, 98]. Although biotype 3 strains have been reported to cause wound infection in humans, to date these infections have been limited to persons handling fish (genus *Tilapia*) in Israel at freshwater fish farming sites [66, 99]. Thus, we may not have obtained any biotype 2 or 3 strains because none of our environmental strains were obtained from such specialized environments.

However, the use of 'biotyping' in the taxonomy of VV is somewhat controversial. Recently, Broza [100] challenged the classification of VV strains by the biotype scheme because of the ambiguity of this scheme as observed by Biosca et al. [101] for indole-positive, eel-infecting VV strains. Although indole, ODC, and ONPG findings are certainly useful to some extent and are still widely used for taxonomic tests to differentiate sub-species groups of VV, we agree that classification systems of VV based on only three enzymatic reactions might not properly reflect the exquisite taxonomic patterns of VV. Nonetheless, the scope of our study was somewhat conservative in that it was confined to VV biotype 1. Thus, we recognize that our results might not adequately reflect the entire span of genetic diversity or population structure of VV.

### BOX-PCR

**Fig 1:**
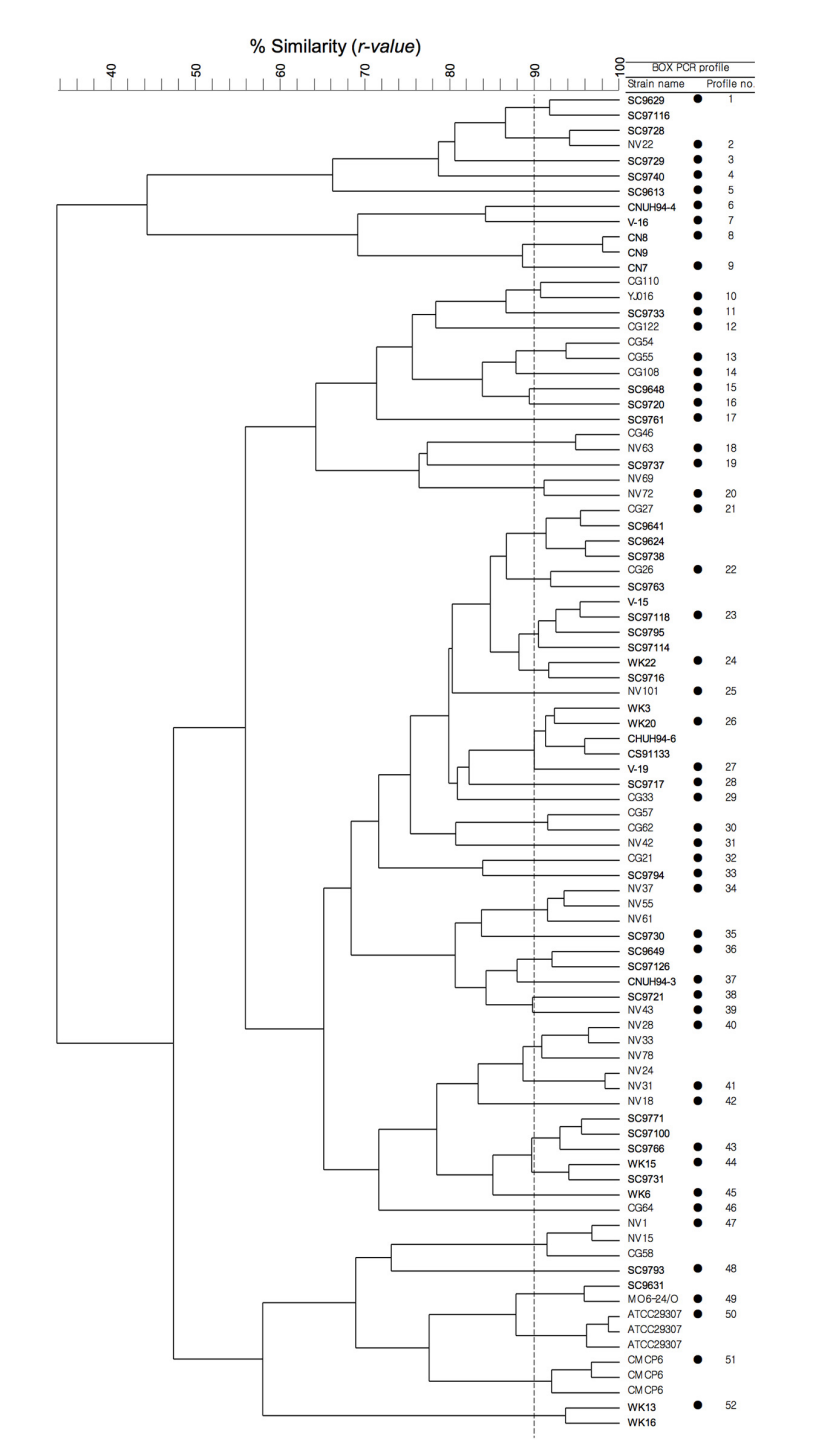
UPGMA cluster analysis of BOX-PCR genomic fingerprints of *Vibrio vulnificus* strains. *r* values are expressed as percentages. The dashed line indicates the cutoff level (90%). Closed circles denote representative strains selected from each of the unique fingerprinting profiles for subsequent analyses.

Prior to in-depth analyses, the VV strains were subjected to BOX-PCR genomic fingerprinting to identify clonal strains (VV strains with identical BOX-PCR fingerprinting patterns) in our collection (Fig 1). To determine a cut-off value to define a unique BOX-PCR fingerprinting pattern (BOX-PCR type), two strains (CMCP6 and ATCC 29307) were subjected to multiple rounds of BOX-PCR. A comparison of the resulting fingerprinting patterns resolved on independent gels yielded an average similarity coefficient (*r* value) of 0.96 (range, 0.92–0.98), which is consistent with other studies [68, 70, 102]. Thus, a similarity value of 0.90 or more was chosen to indicate strains of the same BOX-PCR type. The cut-off level used in the UPGMA cluster analysis of BOX-PCR genomic fingerprinting patterns corresponded to a genomic DNA-DNA similarity of > 95% [103], suggesting that strains belonging to the same BOX-PCR type in this study can be regarded as nearly identical strains at the genomic DNA level.

Cluster analysis identified a total of 52 BOX-PCR types (Fig 1 and Table 2); the majority (58%) of these were singletons (one member strain per BOX-PCR type), indicating that the intra-species diversity of VV as assessed by BOX-PCR fingerprinting could be higher than that observed in this study (Fig 1 and Table 2). Rarefaction analysis of the BOX-PCR results using the Chao-1 estimator [104, 105] suggested that there could be around 30 additional BOX-PCR types (Chao-1 estimate, 84.7). Based on the numbers of identified BOX-PCR types, the environmental VV strains (41 BOX-PCR types) seemed to be more diverse than the clinical strains (16 BOX-PCR types). However, this simple comparison might not be sufficient to draw an adequate conclusion regarding the relationship between diversity and strain source, since three times more environmental strains than clinical strains were included in our collection. Moreover, the evenness indices (J, normalized form of diversity index [0–1 scale] based on Shannon's entropy H) [106] for the environmental strains and clinical strains were 0.868 and 0.870, respectively (for all strains, J = 0.864), indicating that the diversity of the clinical strains as revealed by BOX-PCR fingerprinting was comparable to that of the environmental strains.

### Associations between isolation source and genotypic characteristics

To determine the genotypes of our VV strains, 52 representative strains of BOX-PCR types were subjected to PCR-based genotyping assays. These assays detected polymorphisms at seven genetic loci (the 16S rRNA gene and the *pilF*, *vcg*, *viuB*, *vuuA*, *vvhA*, and CPS alleles) (Table 2) suggested to be predictive of pathogenicity (with the exception of *vvhA*). All VV strains examined in this study were positive for *vvhA*. Since *vvhA* has been shown to be carried by all VV strains isolated to date and could be a hallmark of the VV species itself [107], this gene was excluded from further analysis. Specifically, the 16S rRNA genotype B, CPS genotype 1, *vcg* genotype C, and positive PCR results for *pilF*, *viuB*, and *vuuA* have all been reported to be associated with pathogenicity [30, 37, 71–75]. Thus, these genotypes were designated as 'P' genotypes in the present study. In contrast, the alternative genotypes for these loci (16 rRNA genotype A, CPS genotype 2, *vcg* genotype E, and negative PCR results for *pilF*, *viuB*, and *vuuA*) were all designated as 'N' genotypes. Although a single genotype was not assumed to indicate the virulence potential of VV, our designations (P vs N genotypes) have an advantage over the original designations (e.g., A or B, 1 or 2, C or E, positive or negative) in that they provide an unified designation scheme with an indication (P, pathogenic; N, nonpathogenic) of the possible relationship to the virulence potential of VV strain based on the current knowledge.

The majority (> 50%) of the VV strains in our collection were found to have P genotypes for all loci tested (16S rRNA type B, 88.5%; CPS type 1, 51.9%; *vcg* type C, 90.4%; *pilF* +, 88.5%; *viuB* +, 59.6%; *vuuA* +, 62.2%). No hybrid ribotypes for the 16S rRNA genotype (type AB) [108] were observed. The CPS genotype could not be determined for 10 strains (19%) because no PCR amplification products were obtained using the primer pairs suggested by Han et al. [107]. As reported in numerous studies [30–36], all clinical strains in our collection exhibited P genotypes for the 16S rRNA gene, *pilF*, *vcg*, and *vuuA*. However, some (40% and 20%) of the clinical strains exhibited N genotypes for *viuB* and CPS, respectively. This finding is particularly interesting because even the well-studied clinical strains CMCP6 and YJ016 exhibited N genotypes for these two loci. In contrast, the clinical strain MO6-24/O exhibited P genotypes for all loci tested. Another noteworthy finding was that many of the environmental strains tested exhibited P genotypes for a number of loci. Less than half of the environmental strains exhibited N genotypes for *viuB* and *vuuA* loci (40.5% and 43.2%, respectively), and only a small fraction of the environmental strains exhibited N genotypes for the 16S rRNA gene (16.2%), CPS (32.4%), *pilF* (16.2%), and *vcg* (13.5%).

Although many studies have reported that VV genotype correlates with strain origin (i.e., P genotypes with clinical sources and N genotypes with environmental sources) [32, 34, 37, 71, 72, 108–111], our genotyping findings contradict this conclusion. Statistical analysis using the chi-square (χ^2^) test (α = 0.05) indicated that the association between genotype and strain source was not significant for any genotype, with the exception of *vuuA* (Table 3). For *vuuA*, Fisher's exact test also failed to reject the null hypothesis of random association (*p* < 0.05). While a significant association (*p* < 0.05, χ^2^ test; *p* < 0.05, Fisher’s exact test) was observed between the *vuuA* genotype and the strain origin, the degree of association expressed as a simple matching coefficient indicated that only around 60% of all VV strains showed the expected association (*vuuA-*P types from clinical sources and *vuuA*-N types from environmental sources). A marginal association (*p* = 0.097) was observed for the 16S rRNA and *pilF* genotypes, with degrees of association < 50%. Our results are consistent with those of a recent study of VV strains isolated in Japan [38]. In this study, P genotypes for the 16S rRNA gene, CPS, and *vcg* dominated both in clinical strains and in environmental strains. In addition, the prevalence of the P genotype for the 16S rRNA gene in environmental VV strains isolated in Korea has also been reported [112]. The genotypes of VV might exhibit distinct geographical patterns of distribution, as noted by Reynaud et al. [36]. This hypothesis could explain the discrepancy between our results and those of previous studies; however, this hypothesis still requires further testing.

**Table 3:** Associations between *Vibrio vulnificus* strain origin (clinical or environmental) and genotype.

Gene^a^	χ^2^ test *p* value	Fisher's exact test *p* value	Association index (simple matching coefficient)
16S rRNA	0.097	0.165	0.404
CPS	0.113	0.180	0.571
*pilF*	0.097	0.165	0.404
*vcg*	0.134	0.305	0.385
*viuB*	0.971	≈ 1	0.462
*vuuA*	**0.002** ^b^	**0.002**	**0.596**

^a^ Genes used for genotyping.

^b^ Significant associations are designated in bold.

### Associations among genotypic characteristics

We next determined whether the genotypes of different loci correlated with one another. Significant (*p* < 0.05, χ^2^ test; *p* < 0.05, Fisher’s exact test) associations were observed between the genotypes of all tested loci, with the exception of CPS (Table 4 and S2 Table). The inter-relationships between the 16S rRNA gene, the *pilF* genotype, and the *vcg* genotype were particularly strong. Strikingly, the genotypes of these three loci agreed more than 90% with each other. Consistent with our results, a significant association between the 16S rRNA gene and the *vcg* genotype was reported in a study by Thiaville et al. [34], which was also performed with biotype 1 strains. In their study, 16S rRNA type A and *vcg* type E strains were defined as 'profile 1' strains, whereas 16S rRNA type B and *vcg* type C strains were defined as 'profile 2' strains. They concluded that profile 2 strains were more likely to cause lethal systemic infection, but suggested that genotype alone could not sufficiently predict the pathogenicity of a given VV strain because of the many observed exceptions (e.g., nonpathogenic strains with profile 2). In our study, all clinical strains were profile 2 strains; however, most environmental strains (83.8%) were also profile 2 strains.

**Table 4:** Associations between genotypic characteristics (lower left half, χ^2^ test *p* value; upper right half, simple matching coefficient).

Gene^a^	16S rRNA	CPS	*pilF*	*vcg*	*viuB*	*vuuA*
16S rRNA		0.690	**0.923** ^**b**^	**0.981**	**0.712**	**0.769**
CPS	0.052		0.643	0.690	0.595	0.643
*pilF*	**< 0.001**	0.666		**0.904**	**0.673**	**0.808**
*vcg*	**< 0.001**	0.052	**< 0.001**		**0.692**	**0.750**
*viuB*	**0.002**	0.495	**0.023**	**0.004**		0.596
*vuuA*	**0.003**	0.430	**< 0.001**	**0.012**	0.346	

^a^ Genes used for genotyping.

^b^ Significant associations are designated in bold.

### Multilocus sequence typing (MLST)

MLST of our VV strains revealed 23–35 (average, 28) allelic types (ATs) per locus analyzed (S1 Fig and S3 Table), resulting in a total of 44 unique combinations of ATs. Each unique combination of ATs was defined as an MLST sequence type (ST) in this study (S3 Table). The nucleotide diversity (π) of each gene (average, 0.021; range, 0.015–0.030) (Table 5) was comparable to MLST data previously obtained for pathogenic bacteria, including VV [36, 95, 113–116]. Among the genes analyzed, the highest nucleotide diversity was observed for *pyrC* (π = 0.030), which also contained the largest number (n = 71) of polymorphic sites (Table 5). On the other hand, *recA* showed the lowest nucleotide diversity (π = 0.015), even though it had the largest number (n = 35) of ATs. Despite its low nucleotide diversity, the high allelic diversity of *recA* can be explained by the even distribution over many polymorphic sites of a relatively small number of nucleotide substitutions in the *recA* sequence, which also resulted in *recA* having the highest number of singleton ATs. The dN/dS ratios for all genes analyzed were much lower than 1 (average, 0.010; range, 0.001–0.020), indicating that synonymous substitutions predominated over nonsynonymous substitutions (Table 5). Thus, we conclude that negative (purifying) selection acted against amino acid substitutions in these genes (negative selection on the protein level). This conclusion was expected, because six of the seven genes analyzed by MLST were functionally constrained housekeeping genes. A putative virulence-associated gene, *vvhA*, was also shown to be under negative selection pressure (dN/dS = 0.009). The Tajima's D values for all genes analyzed fell within a confidence interval of zero (e.g., -1.8 < 95% CI < +2.0 and -1.6 < 90% CI < +1.7 for 28 ATs), under the assumption that the Tajima's D values followed a β distribution [85], and hence were considered insignificant (Table 5). Thus, mutations on the DNA level with no effect on fitness can explain most of the observed polymorphisms. In fact, as expected from the very low dN values (0.000–0.002), the amino acid sequences encoded by each gene showed at least 99.5% pairwise identity.

**Table 5:** Characteristics of the genes analyzed by MLST.

Group	Gene	No. allelic types (ATs)		No. of polymorphic sites	No. of parsimonious site	Average nucleotide diversity (π^a^)	dN^b^	dS^c^	dN/dS	Tajima's D^d^
		Total	No. of singletones							
Total	*glnA*	21	12 (57%)	30	19	0.020	0.001	0.081	0.007	-0.200
	*glp*	27	16 (59.3%)	53	43	0.023	0.001	0.097	0.008	0.244
	*gyrB*	25	10 (40%)	38	26	0.018	0.001	0.075	0.011	0.180
	*mdh*	23	13 (56.5%)	35	25	0.016	0.001	0.058	0.011	0.299
	*pyrC*	32	24 (75%)	71	53	0.030	0.002	0.116	0.020	0.413
	*recA*	35	23 (65.7%)	59	40	0.015	0.000	0.061	0.001	-0.455
	*vvhA*	23	10 (43.5%)	36	31	0.024	0.001	0.101	0.009	0.674
	Average	26.6	15.4 (57.9%)	46	33.9	0.021	0.001	0.084	0.010	
Lineage A	*glnA*	4	2 (50%)	5	0	0.006	0.000	0.026	0.000	-0.797
	*glp*	6	6 (100%)	15	7	0.010	0.000	0.044	0.000	-0.034
	*gyrB*	5	4 (80%)	9	2	0.007	0.000	0.030	0.000	-0.526
	*mdh*	4	3 (75%)	14	1	0.011	0.001	0.039	0.026	-0.624
	*pyrC*	6	6 (100%)	11	3	0.007	0.001	0.026	0.026	-0.440
	*recA*	6	6 (100%)	14	4	0.007	0.000	0.027	0.000	-0.666
	*vvhA*	4	3 (75%)	5	1	0.006	0.003	0.015	0.180	-0.212
	Average	5	4.3 (86%)	10.4	2.6	0.008	0.001	0.030	0.033	
Lineage B	*glnA*	17	10 (58.8%)	19	15	0.015	0.000	0.062	0.006	0.266
	*glp*	20	9 (45%)	40	28	0.017	0.001	0.071	0.013	-0.318
	*gyrB*	19	5 (26.3%)	26	15	0.012	0.000	0.052	0.004	-0.475
	*mdh*	19	10 (52.6%)	26	19	0.014	0.000	0.054	0.004	0.461
	*pyrC*	25	17 (68%)	58	36	0.022	0.003	0.082	0.031	-0.320
	*recA*	28	16 (57.1%)	41	23	0.009	0.000	0.038	0.003	-1.005
	*vvhA*	18	6 (33.3%)	22	19	0.015	0.001	0.064	0.008	0.335
	Average	20.9	10.4 (49.8%)	33.1	22.1	0.015	0.001	0.060	0.010	

^a^ Average pairwise nucleotide difference per site.

^b^ Average number of nonsynonymous substitutions per nonsynonymous site.

^c^ Average number of synonymous substitutions per synonymous site.

^d^ No Tajima’s D value was significantly deviated from zero (*p* > 0.10).

**Fig 2:**
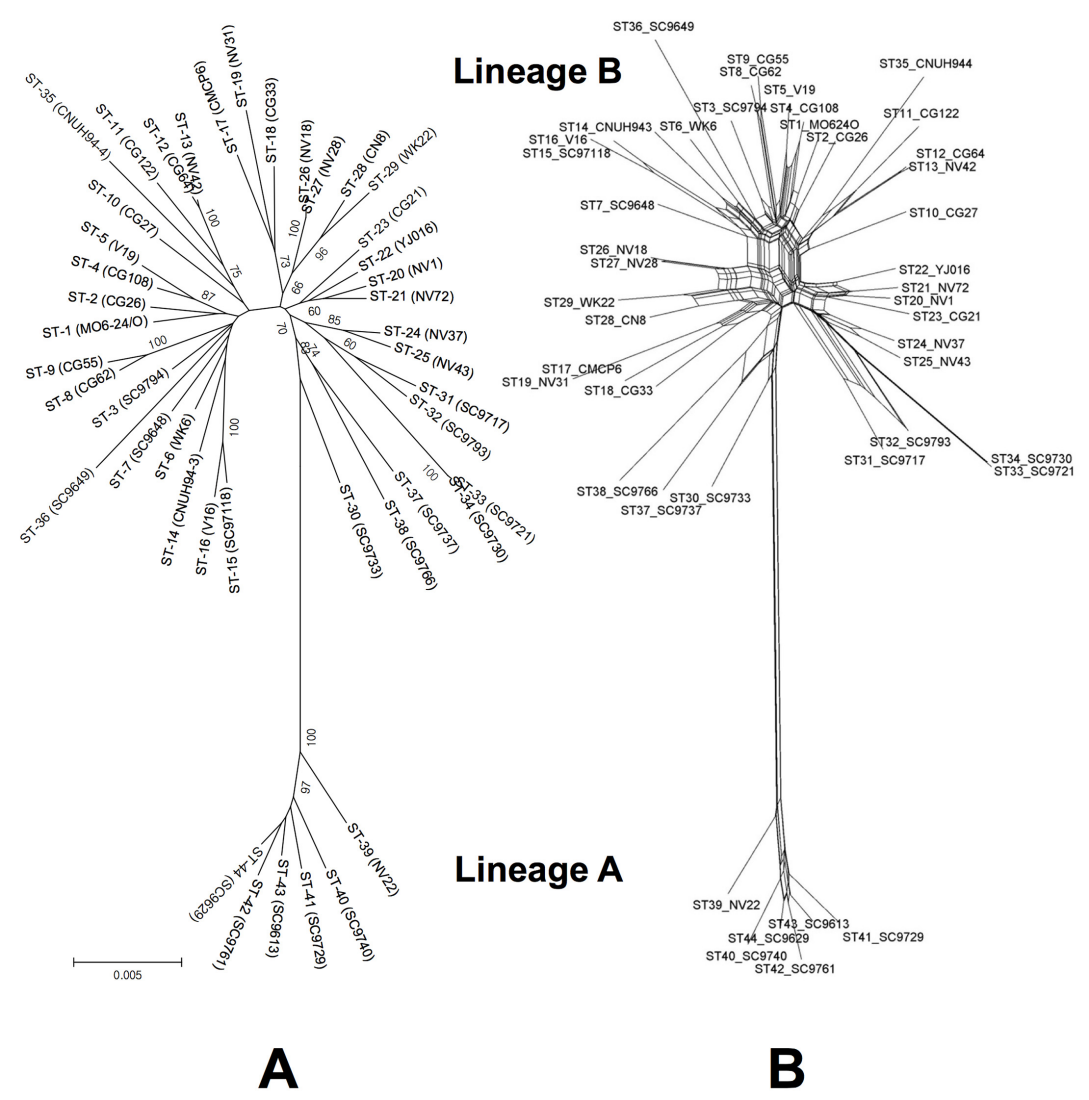
Phylogenetic relationships of *V*. *vulnificus* strains based on the concatenated sequences of seven genetic loci. The phylogenetic distances of each concatenated sequence were calculated using the Jukes-Cantor (JC69) model, and the trees were constructed using the neighbor-joining (NJ) algorithm and the neighbor-net network (NN) algorithm (panels A and B, respectively). The numbers at the nodes in the NJ tree indicate the bootstrap scores (as percentages) and are shown for frequencies at or above the threshold of 50%. Bootstrap scores are not shown in the NN tree for tree legibility, but are given in the text. The scale bar represents the expected number of substitutions per nucleotide position.

When all 44 STs (concatenated sequences) were subjected to phylogenetic reconstruction, two major phylogenetic groups, each of which exhibited a bootstrap confidence level > 80%, were observed in both the NJ tree and the ML tree (Fig 2A). This subdivision of VV into two intra-species lineages is consistent with previous studies [32, 36, 37, 71, 113]. One group was comprised of six STs that originated exclusively from environmental sources, while the other group was comprised of 37 STs that originated either from environmental sources or from clinical sources (Table 1 and S3 Table). We designated the former group as MLST lineage A and the latter group as MLST lineage B; these two MLST lineages corresponded to previously recognized lineages of VV [115] (S2 Fig). We did not consider ST-30 (representative strain, SC9733) as belonging to either of these monophyletic groups because of the low bootstrap confidence level (< 50%) of the internal node of ST-30. In spite of the close relationship between ST-30 (average genetic distance, 0.016 ± 0.002) and the MLST B lineage, no assignment was made for ST-30 in order to avoid making either lineage polyphyletic. As mentioned above for the strain sources in each of the MLST lineages, the association between MLST lineage (A or B) and isolation source (clinical or environmental) was not significant (*p* = 0.146, χ^2^ test; *p* = 0.309, Fisher’s exact test). However, significant (*p* < 0.05, χ^2^ test; *p* < 0.05, Fisher’s exact test) associations were observed between MLST lineage and genotype. Specifically, the N-type and P-type strains predominated in MLST lineages A and B, respectively (Pearson's association coefficient Φ = 0.409–1.000). The MLST A and B lineages were comprised exclusively of N-type strains and P-type strains, respectively, especially for the 16S rRNA gene. The average nucleotide diversity of MLST lineage B (π = 0.015 ± 0.004) was significantly greater than that of MLST lineage A (π = 0.008 ± 0.002) (*p* < 0.05, t-test) (Table 5) as reflected in their phylogenetic branching patterns (Fig 2A). The inter-group genetic distance between lineages A and B (0.033 ± 0.002) exceeded the average nucleotide diversities of both lineages. The average dN/dS ratio of MLST lineage A was slightly higher than that of MLST lineage B (Table 5), although this difference was not significant (*p* > 0.05, t-test). Interestingly, MLST lineage B appeared to be further divided into two sub-lineages, one containing the reference strains CMCP6 (ST-17) and MO6-24/O (ST-1) and the other one containing the reference strain YJ016 (ST-22). However, these sub-groupings exhibited low bootstrap confidence levels (< 50%).

### Linkage analysis

We found that VV was in linkage disequilibrium; that is, VV exhibited a clonal population structure with infrequently recombining 'clonal' STs as opposed to frequently recombining 'sexual' STs. When all 44 STs were tested for linkage disequilibrium between alleles using the index of association (I_A_) [88], both the conventional and standardized I_A_ values were significantly different from zero (I_A_ = 0.643, *p* < 0.05; I^S^_A_ = 0.107, *p* < 0.05), indicating nonrandom association between alleles (linkage disequilibrium) (Table 6). Maynard-Smith et al. described a continuum of bacterial population structures, ranging from strictly clonal (e.g., *Neisseria gonorrhoeae*, I_A_ = 0.04) to panmictic (e.g., *Pseudomonas syringae*, I_A_ = 18.35) [88]. Compared with these data, VV appears to exhibit weakly clonal population structure. However, the degrees of linkage disequilibrium (I_A_) estimated by Maynard-Smith et al. [88] were calculated based on electrophoretic types (ETs), which were determined by multilocus enzyme electrophoresis (MLEE). Since these values ultimately depend on amino acid sequence, the degree of linkage disequilibrium measured for VV in the present study could be underestimated (skewed toward linkage equilibrium). This is because our I_A_ values were calculated based on STs that were determined by MLST, which depends on nucleotide sequence. Boyd et al. [117] reported that changes of around 26 nucleotides were required to influence the electrophoretic mobility of housekeeping enzymes. Despite this underestimation, the I_A_ for VV determined in the present study was significantly deviated from zero. We hypothesize that VV would exhibit a highly clonal population structure if its I_A_ value was determined from ETs, because only a very limited number of ETs are available for each locus due to the high level of amino acid sequence identity between ATs (99.8–100%, almost fixed alleles on the protein level).

**Table 6:** Multilocus linkage disequilibrium analysis of *Vibrio vulnificus*.

	V_O_	V_E_	I_A_ (*p* value)	I^S^_A_ (*p* value)
All sequence types (STs)	0.539	0.328	**0.643 (*p* < 0.001)** ^a^	**0.107 (*p* < 0.001)**
Lineage A STs	0.543	0.498	0.091 (*p* = 0.619)	0.015 (*p* = 0.572)
Lineage B STs	0.676	0.425	**0.592 (*p* < 0.001)**	**0.099 (*p* < 0.001)**

V_O_, observed variance of the number of loci at which two STs differ (K) [88].

V_E_, expected variance of K [88].

I_A_, index of association (I_A_ = [V_O_/V_E_]-1) [88].

I^S^_A_, standardized index of association ([I^S^_A_ = I_A_/[L-1], where L = the number of loci) [89].

^a^ Significant linkage disequilibrium groups are marked in bold.

Consistent with our results, Bisharat et al. [115] reported a clonal population structure for VV, although a different association statistic was used for the linkage analysis. A closely related species, *V*. *cholerae*, has also been shown to exhibit a clonal population structure [118] with an I_A_ value (I^S^_A_ = 0.143, p < 0.05) close to that observed in the present study. The clonal population structure observed for the VV STs in the present study could be explained by insufficient recombination between STs, resulting in a high level of association between alleles, as proposed by Maynard-Smith et al. [119]. The rate of recombination of large chromosomal segments might not be sufficient to randomize the genomes or separate the clonal association of the VV strains analyzed in the present study. Interestingly, when the two MLST lineages were considered separately, the STs belonging to MLST lineage A showed no evidence of linkage disequilibrium (I^S^_A_ = 0.015, *p* > 0.05), while MLST lineage B still showed significant linkage disequilibrium (I^S^_A_ = 0.099, *p* < 0.05). However, we cannot exclude the possibility that the linkage equilibrium observed for MLST lineage A might be due to insufficient sampling, as reflected by the high frequency of singleton ATs.

Inconsistent with the observed results of linkage analysis, ancestral recombination events in the genes used in MLST were detected with two different tests for recombination, the PHI test for overall evidence of recombination and Sawyer's runs test for individual recombination events [91, 94] (Table 7). The PHI test detected significant evidence of recombination (permutation *p* value < 0.05) in *glp*, *gyrB*, *mdh*, *pyrC*, and *vvhA* when all STs were tested. The same result was observed for MLST lineage B, but the PHI statistic could not be calculated for a number of genes in MLST lineage A due to the lack of phylogenetically informative (parsimonious) sites (Table 5). Sawyer's test, a more conservative test for recombination events, also detected evidence of recombination for four genes (*glnA*, *glp*, *gyrB*, and *pyrC*) (Table 7). However, breakpoint analysis identified only a limited number of recombination events. Although 1–25 DNA fragments (segments) were shared by paired sequences via ancestral gene conversions, only 1–4 (and usually only one) recombination events were identified within MLST lineage B and between MLST lineages A and B. Moreover, Sawyer's test did not detect any recombination events within MLST lineage A. This finding supports our skeptical view for the linkage equilibrium observed for MLST lineage A.

**Table 7:** Analysis of overall recombination (PHI test) and individual recombination events (Sawyers’ runs test).

Gene	PHI test *p* value			Sawyer's runs test^a^						
	Total	Within lineages		Simulated *p* value^b^	No. of fragments^c^	No. of recombination events^d^				
						Total	Within lineages		Between lineages	Multiple^e^
		A	B				A	B	A-B	
*glnA*	0.213	ND^f^	0.836	0.013	1	1	0	1	0	0
*glp*	**0.001** ^g^	0.004	**< 0.001**	**0.001**	25	1	0	0	0	1
*gyrB*	**< 0.001**	ND	**< 0.001**	0.005	8	1	0	0	1	0
*mdh*	0.041	ND	**0.003**	0.561	0	0	0	0	0	0
*pyrC*	**< 0.001**	1.000	**< 0.001**	**0.002**	12	4	0	3	1	0
*recA*	0.490	0.256	0.901	0.109	0	0	0	0	0	0
*vvhA*	**0.002**	ND	**< 0.001**	0.274	0	0	0	0	0	0
Sum					46	7	0	4	2	1

^a^ Gene conversion test for recombination between ancestors of sequences in an alignment (global inner recombination) [94, 95]. Global outer recombination events were not detected.

^b^ Global *p* value obtained using 10,000 permutations.

^c^ Fragment shared by two sequences in the alignment via ancestral gene conversion [94]. Only fragments that significantly (*p* < 0.05) implied recombination events were counted.

^d^ Groups of fragments linked to the same 5' and/or 3' breakpoints were classified as a single recombination event as described by Nightingales et al. [95] and den Bakker et al. [96].

^e^ Recombination events both between and within lineages.

^f^ Not determined due to the lack of parsimonious sites.

^g^ Significant (*p* < 0.05) support for recombination.

## Concluding Remarks

We hypothesize that the clonal population structure of VV, which comprised STs with some recombination potential, may be influenced by ecological factors that restrict the breakdown of linkage disequilibrium. Consistent with this hypothesis, *V*. *cholerae* and *Haemophilus influenza* have been shown to maintain clonal population structures, even though these organisms are naturally transformable [88, 118, 120]. Alternatively, VV could be comprised of distinct ecotypes [121], between which recombination is limited. According to the ecotype model of bacterial species, in which distinct ecotypes evolve primarily by periodic selection or selective sweep, alleles are likely to be in significant linkage disequilibrium [122]. The two MLST lineages (or certain ST groups) of VV might correspond to two (or multiple) ecotypes of VV, either of which could be a derived/ancestral ecotype. Although Tajima's D values in the present study were not significantly deviated from zero (Table 5), the positive values observed for all VV STs are consistent with this hypothesis because Tajima's D test tends to produce positive values for a sub-dividing population. Neighbor-Net network analysis further revealed that recombination between MLST lineages A and B was relatively rare compared with recombination within MLST lineage B (Fig 2B). Considering the star-shaped topology and the many cyclic branches near the root of MLST lineage B, this lineage may have undergone rapid clonal expansion after a period of frequent recombination. We speculate that MLST lineage B, or at least some STs in MLST lineage B, correspond to the pathogenic lineage (or pathogenic ecotypes) of VV, since all of our clinical strains belonged to MLST lineage B, and P genotypes predominated in MLST lineage B. The predominance of lineage B strains with P genotype characteristics in our strain collection also suggests the prevalence of potentially pathogenic strains in the marine environment in East Asia. However, for this suggestion to be experimentally supported, the pathogenicity of our strains should be determined using an animal model. In addition, a more extensive MLST analysis of VV, incorporating many more genetic loci, would help clarify the population structure of VV. Inferring phylogenetic relationships from the core genome of VV as constructed from both clinical strains and environmental strains would also help meet this goal. Until recently, studies of VV have focused mainly on clinical strains, especially the well-characterized strains CMCP6, MO6-24/O, and YJ016. It is therefore very important for future studies to include a comprehensive group of environmental strains and to integrate in vivo pathogenicity results with genomic data. This approach will enable a better understanding of the evolutionary patterns and pathogenesis of this notorious marine pathogen.

## Supporting Information

S1 Fig Phylogenetic relationships of *V*. *vulnificus* strains based on the 16S rRNA gene (A), *glnA* (B), *glp* (C), *gyrB* (D), *mdh* (E), *pyrC* (F), *recA* (G), and *vvhA* (H). The phylogenetic distances of each concatenated sequence were calculated using the Jukes-Cantor (JC69) model, and the trees were constructed using the neighbor-joining (NJ) method. The numbers at the nodes in the NJ trees indicate the bootstrap scores (as percentages) and are shown for frequencies at or above the threshold of 50%. The scale bar represents the expected number of substitutions per nucleotide position.
(TIFF)

S2 Fig Phylogenetic tree showing that MLST lineages A and B identified in this study correspond to previously recognized lineages of *V*. *vulnificus*. The tree includes five additional reference strains (strains 101/4, 2322, 99–796 DP-E7, ATL6-1306, and ATL-71503) belonging to MLST lineage A in addition to strains in our collection (the strains included in Fig 2). The lineage information and multi-locus (*glnA*, *glp*, *gyrB*, *mdh*, *pyrC*, *recA*, and *vvhA*) sequences of the additional reference strains were obtained from Bisharat et al. (115) and genome sequences deposited in the NCBI GenBank database (GenBank accession numbers JQDT01000016, JQDS01000033, JSWN01000066, JSWO01000069 and JSWP01000065) (124, 125), respectively. All reference strains are marked by closed circles. The phylogenetic distances were calculated using the JC69 model, and the tree was constructed using the NJ method. The numbers at the nodes indicate the bootstrap scores (as percentages) and are shown for frequencies at or above a threshold of 50%. The scale bar represents the expected number of substitutions per nucleotide position.
(TIFF)

S1 Table Genes used for multilocus sequence typing (MLST) of *V*. *vulnificus* strains. (DOCX)

S2 Table Associations between genotypic characteristics (lower left half, Fisher’s exact test p value; upper right half, simple matching coefficient). (DOCX)

S3 Table Allelic profiles of the *V*. *vulnificus* strains selected based on their BOX-PCR results. (DOCX)
