# Supplementary figures and images for: Genotypic Diversity and Population Structure of Vibrio vulnificus Strains Isolated in Taiwan and Korea as Determined by Multilocus Sequence Typing
Source: PLoS One. 2015 Nov 23;10(11):e0142657. doi: 10.1371/journal.pone.0142657 (PMC4658092; doi:10.1371/journal.pone.0142657)

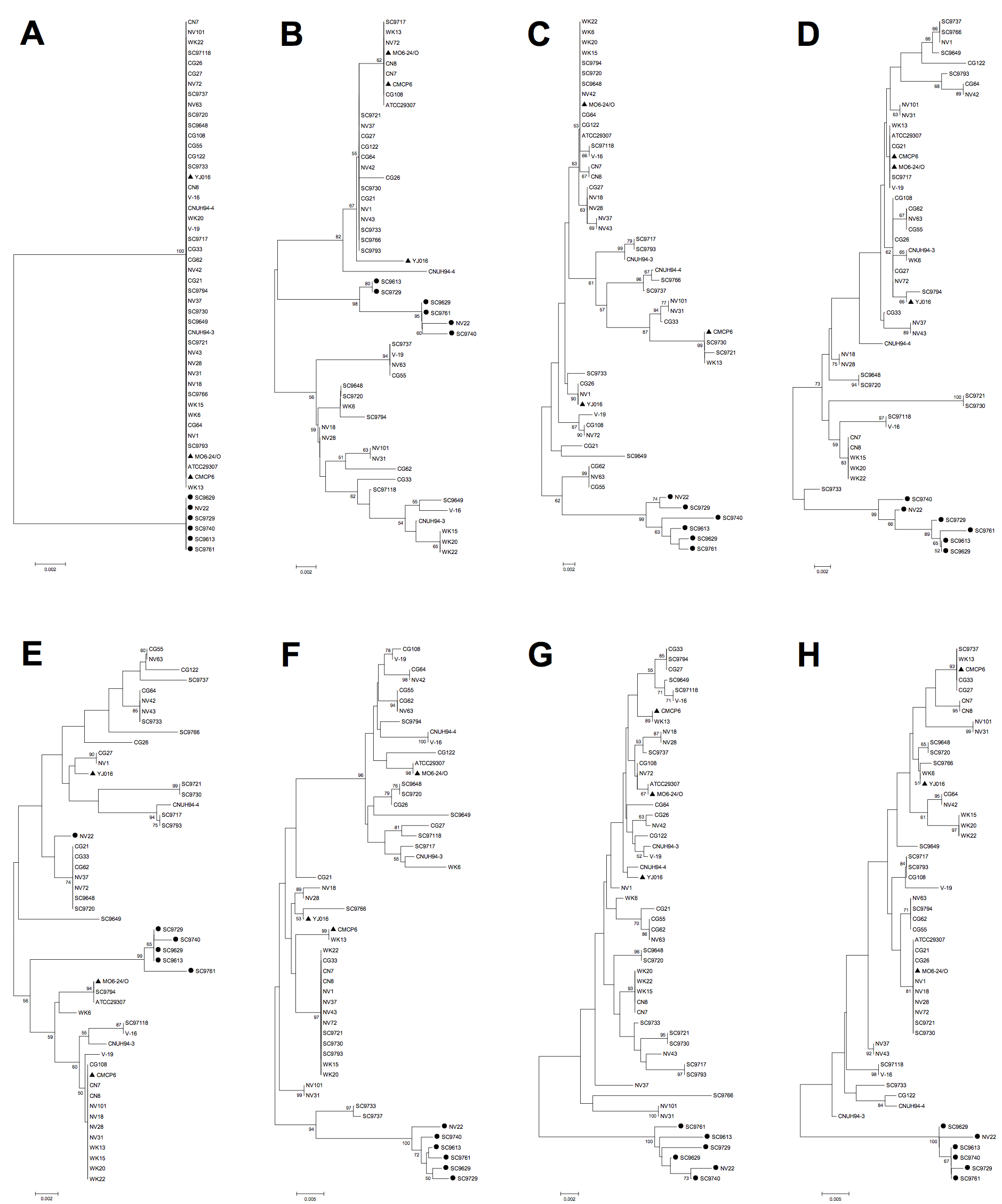

Supplement: S1 Fig — The phylogenetic distances of each concatenated sequence were calculated using the Jukes-Cantor (JC69) model, and the trees were constructed using the neighbor-joining (NJ) method. The numbers at the nodes in the NJ trees indicate the bootstrap scores (as percentages) and are shown for frequencies at or above the threshold of 50%. The scale bar represents the expected number of substitutions per nucleotide position. (TIFF) [file pone.0142657.s001.tiff]

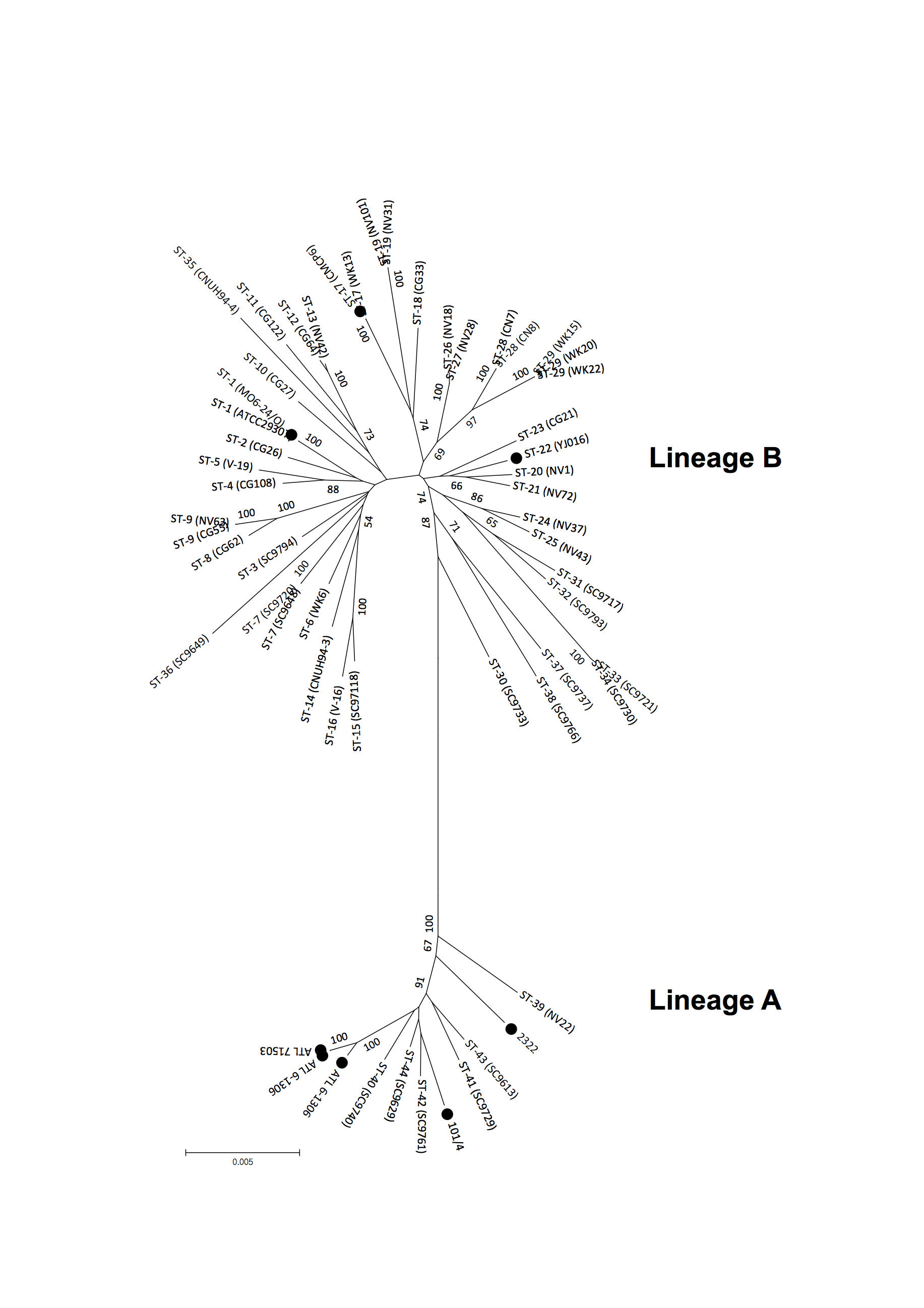

Supplement: S2 Fig — The tree includes five additional reference strains (strains 101/4, 2322, 99–796 DP-E7, ATL6-1306, and ATL-71503) belonging to MLST lineage A in addition to strains in our collection (the strains included in Fig 2). The lineage information and multi-locus (glnA, glp, gyrB, mdh, pyrC, recA, and vvhA) sequences of the additional reference strains were obtained from Bisharat et al. (115) and genome sequences deposited in the NCBI GenBank database (GenBank accession numbers JQDT01000016, JQDS01000033, JSWN01000066, JSWO01000069 and JSWP01000065) (124, 125), respectively. All reference strains are marked by closed circles. The phylogenetic distances were calculated using the JC69 model, and the tree was constructed using the NJ method. The numbers at the nodes indicate the bootstrap scores (as percentages) and are shown for frequencies at or above a threshold of 50%. The scale bar represents the expected number of substitutions per nucleotide position. (TIFF) [file pone.0142657.s002.tiff]
