# Supplementary material for: Genotypic Diversity and Population Structure of Vibrio vulnificus Strains Isolated in Taiwan and Korea as Determined by Multilocus Sequence Typing
Source: PLoS One. 2015 Nov 23;10(11):e0142657. doi: 10.1371/journal.pone.0142657 (PMC4658092; doi:10.1371/journal.pone.0142657)
Supplement: S1 Table — (DOCX) [file pone.0142657.s003.docx]

**S1 Table.** Genes used for multilocus sequence typing (MLST) of *V. vulnificus* strains.

| Gene | Genomic information^a^ | | | | | PCR amplification | | | Sequence fragment used in MLST | | |
| --- | --- | --- | --- | --- | --- | --- | --- | --- | --- | --- | --- |
|  | Chromosome location^b^ | Locus tag | Genomic region^c^ | No. of bases | No. of deduced^d^ amino acids | Primer pair reference | Genomic region^c^ of amplicon | Amplicon size (bp) | Genomic region of fragment | No. of bases | No. of deduced^d^ amino acids |
| *glnA* | Chr-1 | VV1-0889 | 899091-900500 | 1,410 | 470 | Gutacker et al. 2003 | 899399-899836 | 438 | 899418-899819 | 402 (28.5) | 134 |
| *glp* | Chr-1 | VV1-1396 | 1379270-1380922 | 1,653 | 551 | PubMLST | 1379819-1380496 | 678 | 1379843-1380475 | 633 (38.3) | 211 |
| *gyrB* | Chr-1 | VV1-0996 | 1001556-999139^e^ | 2,418 | 806 | Kotetishvili et al. 2003 | 1000902-1000274^e^ | 629 | 1000294-1000884 | 591 (24.4) | 197 |
| *mdh* | Chr-1 | VV1-0673 | 695548-694616^e^ | 933 | 311 | PubMLST | 695404-694703^e^ | 702 | 694727-695383 | 657 (70.4) | 219 |
| *pyrC* | Chr-2 | VV2-1596 | 1753280-1752237^e^ | 1,044 | 348 | PubMLST | 1753157-1752460^e^ | 698 | 1752480-1753136 | 657 (62.9) | 219 |
| *recA* | Chr-1 | VV1-1591 | 1563785-1564834 | 1,050 | 350 | Gutacker et al. 2003 | 1563788-1564668 | 881 | 1563809-1564648 | 840 (80.0) | 280 |
| *vvhA* | Chr-2 | VV2-0404 | 434708-436123 | 1,416 | 472 | Han et al. 2010 | 434748-435266 | 519 | 434768-435247 | 480 (33.9) | 160 |
| Concatenated sequence |  |  |  |  |  |  |  |  |  | 4260 (42.9) | 1420 |

^a^ Based on the genome of *V. vulnificus* CMCP6 (GenBank accession numbers NC_004459.3 and NC_004460.2 for the large and small chromosomes, respectively) [[68](#_ENREF_68)].

^b^ Chr-1 and Chr-2 denote the large and small chromosomes, respectively.

^c^ Genome-based nucleotide positions (start and end).

^d^ *In silico* translation.

^e^ Inverted.
