# Supplementary material for: Genotypic Diversity and Population Structure of Vibrio vulnificus Strains Isolated in Taiwan and Korea as Determined by Multilocus Sequence Typing
Source: PLoS One. 2015 Nov 23;10(11):e0142657. doi: 10.1371/journal.pone.0142657 (PMC4658092; doi:10.1371/journal.pone.0142657)
Supplement: S2 Table — (DOCX) [file pone.0142657.s004.docx]

**S2 Table.** Associations between genotypic characteristics (lower left half, Fisher’s exact test *p* value; upper right half, simple matching coefficient).

| Gene^a^ | 16S rRNA | CPS | *pilF* | *vcg* | *viuB* | *vuuA* |
| --- | --- | --- | --- | --- | --- | --- |
| 16S rRNA |  | 0.690 | **0.923^b^** | **0.981** | **0.712** | **0.769** |
| CPS | 0.122 |  | 0.643 | 0.690 | 0.595 | 0.643 |
| *pilF* | **0.001** | ≈ 1 |  | **0.904** | **0.673** | **0.808** |
| *vcg* | **0.000** | 0.122 | **0.008** |  | **0.692** | **0.750** |
| *viuB* | **0.003** | 0.516 | **0.034** | **0.008** |  | 0.596 |
| *vuuA* | **0.008** | 0.649 | **0.000** | **0.027** | 0.375 |  |

^a^ Genes used for genotyping.

^b^ Significant associations are designated in bold.
