# Supplementary material for: Genotypic Diversity and Population Structure of Vibrio vulnificus Strains Isolated in Taiwan and Korea as Determined by Multilocus Sequence Typing
Source: PLoS One. 2015 Nov 23;10(11):e0142657. doi: 10.1371/journal.pone.0142657 (PMC4658092; doi:10.1371/journal.pone.0142657)
Supplement: S3 Table — (DOCX) [file pone.0142657.s005.docx]

**S3 Table.** Allelic profiles of the *V. vulnificus* strains selected based on their BOX-PCR results.

| BOX-PCR genome fingerprinting | | | Multilocus sequence typing (MLST) | | | | | | | | |
| --- | --- | --- | --- | --- | --- | --- | --- | --- | --- | --- | --- |
| BOX-PCR profile no. | Representative strains | No. of member strians | Allelic type^a^ | | | | | | | Sequence type (ST)^b^ | MLST lineage^c^ |
|  |  |  | *glnA* | *glp* | *gyrB* | *mdh* | *pyrC* | *recA* | *vvhA* |  |  |
| 1 | SC9629 | 2 | 21 | 26 | 25 | 23 | 28 | 34 | 21 | 44 | A |
| 2 | NV22 | 2 | 19 | 23 | 22 | 20 | 27 | 33 | 20 | 39 | A |
| 3 | SC9729 | 1 | 18 | 24 | 24 | 23 | 29 | 31 | 22 | 41 | A |
| 4 | SC9740 | 1 | 20 | 22 | 21 | 22 | 30 | 35 | 23 | 40 | A |
| 5 | SC9613 | 1 | 18 | 25 | 25 | 23 | 31 | 30 | 23 | 43 | A |
| 6 | CNUH94-4 | 1 | 5 | 15 | 9 | 12 | 18 | 4 | 19 | 35 | B |
| 7 | V-16 | 1 | 10 | 2 | 18 | 16 | 18 | 16 | 8 | 16 | B |
| 8 | CN8 | 2 | 3 | 3 | 19 | 19 | 1 | 27 | 9 | 28 | B |
| 9 | CN7 | 1 | 3 | 3 | 19 | 19 | 1 | 27 | 9 | 28 | B |
| 10 | YJ016 | 2 | 4 | 7 | 7 | 2 | 4 | 5 | 13 | 22 | B |
| 11 | SC9733 | 1 | 1 | 6 | 15 | 11 | 9 | 24 | 17 | 30 | ND^d^ |
| 12 | CG122 | 1 | 1 | 1 | 10 | 9 | 17 | 10 | 18 | 11 | B |
| 13 | CG55 | 2 | 12 | 13 | 4 | 10 | 26 | 19 | 2 | 9 | B |
| 14 | CG108 | 1 | 3 | 9 | 6 | 19 | 23 | 1 | 4 | 4 | B |
| 15 | SC9648 | 1 | 17 | 1 | 17 | 1 | 21 | 26 | 11 | 7 | B |
| 16 | SC9720 | 1 | 17 | 1 | 17 | 1 | 21 | 26 | 11 | 7 | B |
| 17 | SC9761 | 1 | 21 | 27 | 23 | 21 | 32 | 32 | 23 | 42 | A |
| 18 | NV63 | 2 | 12 | 13 | 4 | 10 | 26 | 19 | 2 | 9 | B |
| 19 | SC9737 | 1 | 12 | 14 | 12 | 8 | 10 | 2 | 10 | 37 | B |
| 20 | NV72 | 2 | 3 | 9 | 6 | 1 | 1 | 1 | 1 | 21 | B |
| 21 | CG27 | 4 | 1 | 5 | 6 | 3 | 15 | 14 | 10 | 10 | B |
| 22 | CG26 | 2 | 2 | 7 | 6 | 7 | 22 | 8 | 1 | 2 | B |
| 23 | SC97118 | 4 | 9 | 2 | 18 | 16 | 16 | 16 | 8 | 15 | B |
| 24 | WK22 | 2 | 6 | 1 | 19 | 19 | 1 | 27 | 15 | 29 | B |
| 25 | NV101 | 1 | 14 | 18 | 2 | 19 | 7 | 28 | 16 | 19 | B |
| 26 | WK20 | 4 | 6 | 1 | 19 | 19 | 1 | 27 | 15 | 29 | B |
| 27 | V-19 | 1 | 12 | 8 | 1 | 18 | 24 | 12 | 3 | 5 | B |
| 28 | SC9717 | 1 | 3 | 12 | 1 | 13 | 13 | 18 | 4 | 31 | B |
| 29 | CG33 | 1 | 11 | 19 | 3 | 1 | 1 | 14 | 10 | 18 | B |
| 30 | CG62 | 2 | 13 | 13 | 4 | 1 | 26 | 19 | 2 | 8 | B |
| 31 | NV42 | 1 | 1 | 1 | 14 | 11 | 20 | 9 | 14 | 13 | B |
| 32 | CG21 | 1 | 1 | 10 | 1 | 1 | 6 | 20 | 1 | 23 | B |
| 33 | SC9794 | 1 | 16 | 1 | 7 | 14 | 25 | 14 | 2 | 3 | B |
| 34 | NV37 | 3 | 1 | 4 | 8 | 1 | 1 | 17 | 6 | 24 | B |
| 35 | SC9730 | 1 | 1 | 21 | 20 | 4 | 1 | 23 | 1 | 34 | B |
| 36 | SC9649 | 2 | 8 | 17 | 11 | 5 | 11 | 15 | 5 | 36 | B |
| 37 | CNUH94-3 | 1 | 7 | 11 | 5 | 17 | 12 | 11 | 7 | 14 | B |
| 38 | SC9721 | 1 | 1 | 20 | 20 | 4 | 1 | 23 | 1 | 33 | B |
| 39 | NV43 | 1 | 1 | 4 | 8 | 11 | 1 | 25 | 6 | 25 | B |
| 40 | NV28 | 3 | 15 | 5 | 16 | 19 | 3 | 6 | 1 | 27 | B |
| 41 | NV31 | 2 | 14 | 18 | 2 | 19 | 7 | 28 | 16 | 19 | B |
| 42 | NV18 | 1 | 15 | 5 | 16 | 19 | 2 | 6 | 1 | 26 | B |
| 43 | SC9766 | 3 | 1 | 16 | 12 | 6 | 8 | 29 | 12 | 38 | B |
| 44 | WK15 | 2 | 6 | 1 | 19 | 19 | 1 | 27 | 15 | 29 | B |
| 45 | WK6 | 1 | 17 | 1 | 5 | 15 | 14 | 22 | 13 | 6 | B |
| 46 | CG64 | 1 | 1 | 1 | 14 | 11 | 20 | 13 | 14 | 12 | B |
| 47 | NV1 | 3 | 1 | 7 | 12 | 3 | 1 | 21 | 1 | 20 | B |
| 48 | SC9793 | 1 | 1 | 12 | 13 | 13 | 1 | 18 | 4 | 32 | B |
| 49 | MO6-24/O | 2 | 3 | 1 | 1 | 14 | 19 | 3 | 1 | 1 | B |
| 50 | ATCC29307 | 1 | 3 | 1 | 1 | 14 | 19 | 3 | 1 | 1 | B |
| 51 | CMCP6 | 1 | 3 | 21 | 1 | 19 | 5 | 7 | 10 | 17 | B |
| 52 | WK13 | 2 | 3 | 21 | 1 | 19 | 5 | 7 | 10 | 17 | B |
|  | Total | 84 | 21 | 27 | 25 | 23 | 32 | 35 | 23 | 44 | 2 |

^a^ Defined as a unique combination of polymorphisms within a gene. An arbitrary number was assigned to each allelic type.

^b^ Defined by a unique combination of allelic types within a concatenated sequence (seven genes combined).

^c^ MLST lineages were identified from the phylogenetic tree reconstructed from the concatenated sequence. Monophyly of each lineage was supported by bootstrap analysis (bootstrap score > 80%).

^d^ Not determined. No assignment was made due to the low bootstrap score, despite its close relationship to MLST lineage B.
